# Emerging Synergisms Between Drugs and Physiologically-Patterned Weak Magnetic Fields: Implications for Neuropharmacology and the Human Population in the Twenty-First Century

**DOI:** 10.2174/157015907782793603

**Published:** 2007-12

**Authors:** P.D Whissell, M.A Persinger

**Affiliations:** 1 Neuroscience Research Group, Department of Biology, Laurentian University;; 2 Behavioral Neuroscience and Biomolecular Sciences Programs, Laurentian University, Sudbury, Ontario, Canada

**Keywords:** Magnetic field, synergism, nitric oxide, dopamine, serotonin, acetylcholine, opiates, physiologically-patterned.

## Abstract

Synergisms between pharmacological agents and endogenous neurotransmitters are familiar and frequent. The present review describes the experimental evidence for interactions between neuropharmacological compounds and the classes of weak magnetic fields that might be encountered in our daily environments. Whereas drugs mediate their effects through specific spatial (molecular) structures, magnetic fields mediate their effects through specific temporal patterns. Very weak (microT range) physiologically-patterned magnetic fields synergistically interact with drugs to strongly potentiate effects that have classically involved opiate, cholinergic, dopaminergic, serotonergic, and nitric oxide pathways. The combinations of the appropriately patterned magnetic fields and specific drugs can evoke changes that are several times larger than those evoked by the drugs alone. These novel synergisms provide a challenge for a future within an electromagnetic, technological world. They may also reveal fundamental, common physical mechanisms by which magnetic fields and chemical reactions affect the organism from the level of fundamental particles to the entire living system.

## I. INTRODUCTION 

Synergisms, potentiations, and summations between pharmacological agents or endogenous neurochemistry are phenomena that challenge contemporary models and mechanisms and increase the risk of adverse effects from multiple medications. Within the last century and in particular during the last twenty years there have been increasingly prevalent and pervasive stimuli that have the potential to interact with endogenous neurochemistry and pharmacological treatments. These stimuli are environmental electromagnetic fields (MF) whose temporal patterns and frequencies overlap with those generated by living systems. This review examines the available literature on interactions between physiologically-patterned MF and pharmacological agents with particular emphasis on the receptors and neurotransmitters through which these effects might be mediated.

The ubiquity of magnetic fields with extraordinarily complex and potentially physiologically-patterned shapes and intensities is a consequence of a synthetic environment that has been hereto unexperienced by living systems. In addition to the myriad of time-varying patterns generated directly by electronic devices such as computers, light sources, and innumerable household appliances, there are the emergent fields from the rapid growth of communication systems. Although the carrier frequencies are within the GHz range, extraordinary numbers of different signals within this range propagated simultaneously can create a secondary matrix composed of the "beats" or differences between these frequencies. These are within the range generated by the brain.

MF as opposed to electric fields have the capacity to penetrate the volume of matter that defines living systems. Whereas the availability of exogenously applied chemicals to the intracellular environment is contingent upon vasculature and its multiple impedances and filters, appropriately patterned MF have the potential to penetrate homogenously through body space or "focus" upon structures or regions whose intrinsic cohesive temporal patterns of neuroelectrical and electromagnetic activity are systematically related to their own. Whereas models for potential mechanisms were once relegated almost exclusively to some variant of the concept that an induced electric field (and hence current depending upon the local conductivity of the medium) is associated with a changing magnetic field, there are now multiple tested and verified alternative explanations that include the concepts of resonance.

Intensity-dependence in MF research has been considered the powerful analogue of dose-dependence in pharmacological research. Non-linearity in both domains has been accepted and the presence of "bands" or "windows" [1] of maximum biological or biochemical effect as a function of magnetic field intensity or drug concentration are clearly demonstrable [37, 80]. Most conceptual arguments rest upon the assumed magnitude for thresholds. Whereas the empirical observations that a plethora of "maximum" responses that occupy a range from very low (femtomole) to very high (millimole) concentrations of ligands in pharmacology have been resolved by the concept of receptor subtypes, the equivalent has not been seriously considered in magnetobiology and magnetochemistry. Yet reliable biological effects have been reported, depending upon the duration of exposure and the complexity of the magnetic field, with intensities between 1 nanoT and 100 microT (0.1 milliT). This range extends several orders of magnitude and includes most of the values generated within the modern environment.

The interactions between maximally effective applied MF (due to intentional design or by statistical occurrence) and receptor systems stimulated by pharmacological agents could be as powerful as the most potent chemical synergism. In pharmacology, the receptor-agonist interaction within the cell surface ultimately results in a series of intracellular signaling pathways that mediate "the information" afforded by the temporal and spatial patterns of the sequestered neurotransmitter. If this "information," which determines the activity and function of the cell, is a series of frequency-modulated or phase-modulated electromagnetic patterns mediated by calcium waves and related phenomena, then direct interactions with applied MF could occur.

Undertaking the pragmatic development and theoretical understanding of synergisms between weak magnetic fields and pharmacological agents requires an appreciation for complexity. Structure dictates function. Whereas the functions of molecules within biological space are determined by their spatial organization, the functions of weak, physiologically-patterned MF within biological space are determined by their temporal organization. From the perspective of an optimal parsimony, every molecular structure and its interaction with receptors should have an equivalent temporal pattern that can be simulated by the appropriately applied MF.

Until recently the temporal patterns of MF have emphasized the symmetry and simplicity of sinusoidal waves or square waves. Usually intensities (measured in Tesla, T) in the order of milliT or greater have been required to produce reliable effects with these field strengths. On the other hand complex, asymmetrical and physiologically-patterned magnetic fields are effective at intensities many times less (microT to picoT). While large, such ranges are not unusual in neuropharmacology. Whereas extraordinary large amounts of water, a very simple structure, may be required to elicit biological effects, an extremely small amount of a protein, a very complex structure, may be required to produce the same magnitude of effect.

As the frequency of the applied MF determines its physical properties, MF falling within related frequency bands of the electromagnetic spectrum demonstrate some homology in their effects. It is therefore practical to group MF falling within a frequency band into a singular category with distinct characteristics. Here, these categories are defined as extremely low frequency (ELF-MF of ≤ 300 Hz), very high frequency (VHF-MF of 300 MHz – 300 GHz) and static (SMF of effectively 0 Hz or "static"). As investigations into MF focus on the discrete frequencies and frequency bands encountered in occupational and home settings, only a restricted portion of frequencies are explored and here analyzed.

## II. NEUROTRANSMITTER SYSTEMS AND MF 

### a. Opiates

Changes in opiate-mediated behaviours such as nociception are a classic response to ELF- and VHF-MF exposure and have been characterized in many species, including mice [22, 23, 49, 50, 57, 115, 121, 129, 151], rats [6, 25, 48, 85, 117, 162and the land snail,*Cepaea nemoralis* [56, 58, 113,  114, 160, 161, 167]. SMF may have nociceptive and potentially analgesic effects as well [28] but these responses have not been subjected to the experimental rigor as those for ELF- and VHF-MF have been. Alteration in nociception is a robust effect from MF and has obvious therapeutic advantage in the treatment of pain. Not surprisingly, MF-induced analgesia has inspired a large corpus of literature. Collectively, the literature strongly supports that endogenous opiates are among the early effectors of MF-induced analgesia and many other exposure effects, including neurochemical interactions.

The effect of MF exposure on nociception is variable and depends on the field used and species studied. Both augmentation and retardation of analgesia have been demonstrated with simple and complex MF of intermediate intensity (microT to milliT). This bidirectional effect on analgesia may reflect a complex influence on the opiate system. A number of external co-determinants may act in these studies, including levels of light [58, 114]. Though there is a conservation of the structure of the opiate system with evolution [158], differential sensitivity to analgesia-inducing MF exists between species. Shupak and colleagues [151] found no additive analgesic effect between morphine and pulsed MF treatment in CF1 mice, in contrast with past results in *Cepaea nemoralis* shown by Thomas and colleagues. Accordingly, the effect sizes also vary between species, with more robust, strong effects being noted in *Cepaea nemoralis* (50-80%) and mice and weaker effects being noted in rats and humans (10-30%). Effect size is defined here as the amount of variance in the dependent variable explained by the treatment (independent variable) and is equivalent to r-squared values for correlations.

A specific central opiate receptor population is likely involved in all these responses. In exploring the mechanism of this phenomenon, Thomas and others [160] utilized various opiate receptor antagonists to identify the contribution of each subtype of opiate receptor to analgesia. Inhibition of analgesia by physiologically-patterned, frequency-modulated, pulsed MF was seen with the nonspecific antagonist naloxone, mu-opiate receptor antagonists naloxazine and beta-funaltrexamine and delta-opiate antagonists naltrindole-5'-isothiocyanate and ICI 174,864. Antagonism of kappaopiate receptors with nor-binaltorphimine had no effect. In a manner consistent with opiate tolerance, decreases in effectiveness and cross-tolerance of MF-induced analgesia to other opiate agonists developed with repeated exposure [161]. This finding reinforces the conclusion that opiates and MF share a pharmacological substrate within the nervous system. In later work with rats, Lai and Carino [66] showed that the decreases in the cholinergic activity [67] of the hippocampus and frontal cortex that occurred with exposure to a 60 Hz field also involved mu and delta-opiate receptors.

These experiments showed a role of opiates in simple and pulsed ELF-MF response, but opiate-like effects induced by VHF-MF and microwave (MW) fields also exist and have been characterized for more then a decade. Unfortunately, examples have been sparse in recent literature. Pulsed, 2.45 GHz (2 micros pulse width, 500 pulses/s) MW, similar to those emitted by microwave appliances, have been shown to have effects similar to stress-induced states [71] that are sensitive to pharmacological modulation of opiate receptors [65, 69, 70]. Pretreatment with naltrexone reversed many of the effects of MW exposure, including those on cholinergic activity in the hippocampus and frontal cortex [70]. Specific receptor subtypes involved in MW responses have been implicated. Inhibition of the mu-opiate receptors with betafunaltrexamine in the septal nucleus blocked the cholinergic effect of MW [72]. Cholinergic changes within the frontal cortex also resulted from MW exposure; however they were non-responsive to opiate pharmacology and may be dissociated from the central opiate system [69].

The effects of other VHF-MF fields on opiate activity parallel those of ELF-MF. The application of VHF-MF to the skin, which is the basis of millimeter-wave therapy (MWT), may be effective in treating pain [132, 168] and possibly cancers such as melanoma [121]. MWT effects are also highly consistent with and sensitive to opiate modulation. In mice, naloxone pretreatment blocked the analgesia [129], the inhibition of the scratching caused by pruritogenic agents [130], and the lengthening (by 50%) of the anaesthesia evoked by either chloral hydrate (450 mg/kg) or ketamine (80 mg/kg) [131] with MWT. While central opiates appear involved, research has also demonstrated a striking role of the peripheral nervous system as well. Innervation density of free nerve endings within the exposed area determines the effectiveness of MWT [123] and transection of the sciatic nerve abolishes the beneficial effects [122]. Other factors, including the biochemical composition of the tissue, may affect absorption and response [34]. With MWT, central opiates may act in concert with other peripheral magnetosensitive agents to produce the therapeutic outcome.

### b. Serotonin (5-HT) 

Many of the psychological effects of MF, such as the reduction in anxiety and depression seen with repetitive transcranial magnetic stimulation (rTMS) treatment [10, 53, 54] and with ELF-MF [5], are consistent with serotonin (5-hydroxytryptamine or 5-HT) modulation. As well as having unique cognitive effects, 5-HT modulation with MF may contribute to MF-induced analgesia indirectly. Here the relevant effects on 5-HT from ELF-MF and SMF applications, along with the most recent effects identified in rTMS, are discussed.

There are ties between MF-induced 5-HT modulation and MF-induced behavioral changes. Recently, using a 55.6 Hz, 8.1 milliT MF, Bao and colleagues [6] showed an increase in brainstem 5-HT as well as hypothalamic beta-endorphin and substance P in the rat following exposure for 4 days at 6 hr per day. Analgesia exhibited a similar time course and was found on days 3 and 4 only. The conspicuous increase in 5-HT and other biochemical markers was absent past a seventh day of exposure, but the mechanisms of this adaptation were difficult to discern. Down-regulation of 5-HT_2_ receptors is known to occur in the frontal cortex with MF exposure [10] and is accompanied by an up-regulation in beta-adrenergic receptors. Weak, pulsed ELF-MF may be useful in reversing the progressive neuronal atrophy of multiple sclerosis [141] by affecting 5-HT.

In the above studies, the conferred change was short-term and required acute exposure (approximately 1 ksec to 20 ksec). Rajendra and colleagues found no effects on 5-HT in chick embryos exposed for as long as 15 days to a 50 Hz MF [124] while Margonato and others showed no 5-HT effects from a 50 Hz MF applied for 22h/day for 32 weeks [84]. The intensities for both experiments ranged between 5 microT to 0.1 milliT. Significant alterations in hypothalamic 5-HT have been observed in male rats exposed to a 0.1 mT SMF for 1 month [14] but when the exposure period was extended to 4 months no changes were found. No effects have been found with exposure to stronger SMF (80 milliT and 7 T) [64]. In eliciting 5-HT effects, applying the optimal temporal pattern appears more important then the field's intensity. As is found in studies examining the influences of MF on opiate-mediated behaviour, measurements after long exposure periods usually yield negative findings. The "window of influence" [1] for MF upon 5-HT appears short.

The mechanisms of adaptation by the 5-HT system during exposures to MF have only begun to be uncovered. Desensitization of the 5-HT1B receptor following exposure to a 50 Hz, 2.5 milliT MF has been noted [86]. Massot *et al.* [86] hypothesized this change was a primary source of the clinically beneficial effects of rTMS upon depression. Effects on opiate receptor sensitivity were suggested but were not found at the level of significance (p< .001) employed. These results were later independently reproduced using a 50 Hz, 1.1 milliT ELF-MF [30]. In both cases, the effect resulted from acute (about 0.5-3 ksec) exposure.

Many time-varying MF appear to have the potential to influence behaviour through 5-HT, but the number of studies examining this possibility are few as the focus is usually upon opiates. Application of specific and nonspecific antagonists in MF studies would serve to isolate the contribution of 5-HT, if any, to MF-induced behavioral changes.

### c. Dopamine (DA) 

MF effects on dopamine (DA) also present strong therapeutic potential and are thought to occur in rTMS [55] and in the treatment of debilitating disorders such as Parkinson’s disease, a disorder of DA depletion, with ELF-MF [135, 136, 139, 142, 143]. DA modulation may also play a role in the locomotor responses that occur with MF exposure [112]. Many effects on DA pharmacology have been reported which suggest that it plays a strong role in MF responses.

Work by Sandyk and colleagues [135, 136, 139, 142, 143] has repeatedly shown the ability of ultra-weak (pT) ELF-MF to ameliorate the symptoms of Parkinson’s disease and other disorders, such as Tourette's syndrome [137], in a manner consistent with improved DA transmission. In most cases, the D2 receptor is implicated. While this theory has not been verified neurochemically, MF are known to influence transmission at other DA receptors. With exposure to a milliT range, 10 Hz MF, reduction in the reactivity of central D1 receptors may occur [153]. A further consequence of exposure to ELF-MF may be an increase in DA turnover in the frontal cortex [154].

While effects on DA production were not found in these studies, they have been noted elsewhere. In a cellular model of rTMS, Shaul and colleagues [148] reported a decrease in intracellular DA and DA precursor 3,4-dihydroxy-L-phenylalanine (L-DOPA) within neuroblastoma cells following application of a 3 Hz, 1.7 T MF for 10 s. Chronic treatment or use of other frequencies (15, 20, 45 Hz) did not yield an effect. It should be noted that while the application of rTMS is remarkably similar to the application of extracranial ELF-MF by Sandyk, the intensity used in rTMS is greater by a factor of a trillion. The two effects may not necessarily be related. Changes in DA concentration have also been noted with *in vivo* models of rTMS in the area of the dorsal hippocampus [59].

The DA system, in contrast to that of 5-HT, does not appear susceptible to power frequencies (ELF-MF of 50 or 60 Hz). While the intensity of the MF used in these studies varies, very low frequency (< 15 Hz) and short exposure duration (< 3 ksec) appear common and may be critical to the induction of DA effects. The involvement of multiple receptor subtypes of DA with different MF configurations suggests a gradient of DA responses is possible. It should be noted that the beneficial effects found by Sandyk and Iacono were blocked by pre-treatment with naltrexone [143] which strongly suggests, as in the case of MF-induced cholinergic and 5-HT effects, that opiate transmission is involved in their generation.

### d. Nitric Oxide 

The discovery that pharmacological modulation of NO could be involved in the effects of MF [8, 25, 50, 56, 58, 87, 97, 106, 146, 147, 174, 175] has revolutionized the field of bioelectromagnetics. Given the involvement of NO in a multitude of essential biological functions, modulating NO activity with MF has tremendous therapeutic potential.

Studies showing relationships between NO generation and MF exposure have accumulated rapidly over the last few years. Enhancement of NO generation has been seen with application of mT range SMF for 30 min in humans [155], 60 Hz MF for 48 hr in mice [50] and 50 Hz MF in rats [46]. The pattern of NO production across the cerebrum found by Jelenkovic and colleagues in part resembles that of the change in the activity of the cholinergic system demonstrated by Lai and others [66] in that it occurs within the frontal cortex and hippocampus. Exposure to a 0.1 milliT, 60 Hz MF enhanced the NO generation of lipopolysaccarides *in vivo* [175]. Enhanced expression of neuronal nitric oxide synthase (nNOS) a subtype of the NO synthesizing enzyme nitric oxide synthase (NOS) has been noted following exposure to pulsed MF [62].

In contrast with these findings, Noda and colleagues report changes in NO activity of the rat cerebellum with pulsed DC MF [100] but found no effect from AC or static DC MF. Positive NO responses have also been shown for higher frequency ranges. VHF-MF can influence NO activation in the nasal and sinus mucosa [174] and NO production in the cerebellum [97]. NO responses appear to occur at many frequencies and intensity levels and may be, like modulation of opiate transmission, a general correlate of MF exposure. Predicting the direction of NO modulation based on field parameters presents a challenge to future researchers.

The effect sizes in these experiments were relatively strong (15-40%) which suggests a significant relationship between NO and MF exposures that is not causal but involves two or more intervening variables. While there is strong support for a role of NO in many MF-induced responses, direct biomolecular connections between these changes, NO, and NOS subtypes have proved elusive. Additionally, it is sometimes difficult to relate biochemical changes directly to the behavioral effects of MF exposures.

## III. INTERACTIONS BETWEEN ELF AND DRUGS 

### a. Extremely Low Frequency Field (ELF) Interactions with Drugs 

Following public concerns about the health risks of AC power transmission, which utilizes 50 Hz in Europe and 60 Hz in North America, there has been continual research into the effects of power frequency fields on biology and behaviour [31, 116]. The ubiquity of power frequency fields mandates comprehensive investigation into their safety; accordingly research concerning the adverse biological effects of power frequency MF is a broad field and constitutes the core of contemporary MF research in North America and Europe.

While power frequency fields are known to have replicable effects on human biology and behaviour [18, 51, 152], other ELF-MF have significant effects on the organism and should not be overlooked. Square-wave 0.5 Hz [87, 101], 1 Hz [8], and 7 Hz [63, 111] MF, among others, are known to be effective in the experimental setting. Complex MF, which incorporate many different frequencies per complete pulse, have diverse effects and have been shown to effect hippocampal cell morphology [157] and prenatal development [87, 91]. MF modeled after the oscillations of endogenous processes and geomagnetic events are an emerging trend in laboratory research. These "rationally designed" MF are thought to influence biological systems of matched frequency [87]. While ELF-MF and MF in general may not have carcinogenic influences on their own, they may interact with carcinogenic agents [81].

Most investigations into the interaction between ELF-MF and drugs focus on enhancing the pharmacological treatment of pain. NO modulation is one route being investigated as there are multiple synergies between NO modulators and these fields. NO-releasing agents such as s-nitro-n-acetylpenicillamide (SNP) may potentiate the hyperalgesia induced by 60 Hz fields in *Cepaea nemoralis* while nonspecific NO inhibitors such as NG-nitro-l-arginine methyl ester (L-NAME) may suppress it [56]. It is noteworthy that these NO-modulating agents had no effect without MF. In contrast, Dixon and Persinger [25] found that co-administration of L-NAME and amygdaloid-simulated, burst-firing MF counteracted the analgesic effects of morphine on hotplate analgesia. As the influence of MF on analgesia is bidirectional and variable depending upon the experiment, the effect of NO modulation is not always consistent.

Adrenergic receptors may be relevant to ELF-MF-induced analgesia as they can influence the effects of opiate-mediated analgesia [85]. In their experiment, Martin and Persinger demonstrated the ability of central alpha2 adrenergic agonist clonidine to potentiate ELF-induced analgesia. No effect was noted for alpha1 adrenergic antagonist prazosin. These relationships need to be explored in therapeutic settings.

Recently, synergies with clonidine were found to exist for other behaviours. Potentiation of clonidine-induced sleep in 2 day old chicks was reported for ELF-MF [96]. This increase involved gamma amino butyric acid subtype A (GABAA) and benzodiazepine receptors. Coupled with the results and Martin and Persinger [85] these data show that synergies may be bidirectional in that MF and pharmacology have the ability to influence each other’s effects.

Benzodiazepine receptors also appear involved in the hyperalgesic effects of 60 Hz MF on mice [49]. In their experiment, Jeong and others noted that inhibition of benzodiazepine receptors with central competitive antagonist flumazenil abolished the hyperalgesia effect while the application of benzodiazepine agonist diazepam potentiated it. In a more recent study, antagonism of benzodiazepine but not GABAA or GABAB receptors blocked this hyperalgesic effect [48].

ELF-MF may also have important treatment value for partial complex (limbic) epilepsy [2] that can be tested experimentally. In a model of ion cyclotron resonance [78] for lithium, McKay and Persinger [88] showed a decrease in seizure onset time in lithium-pilocarpine seized rats with the application of a field tuned to resonate with lithium ions. The authors noted that these effects could also be consistent with modification of both NO or opiate transmission by MF. The ELF-MF sensitivity of lithium-pilocarpine seized rats given noncompetitive n-methyl-D-aspartate (NMDA) antagonist ketamine post-seizure to prevent morbidity has been shown [90]. Rats administered ketamine (100 mg/kg) immediately after the induction of the lithium-pilocarpine seizures, who are otherwise similar to non-seized controls [169], performed abnormally on a radial arm maze task only when they were exposed to complex, physiologically-patterned magnetic fields with intensities between 7 nT and 500 nT. The latent behavioral deficits suggest a complex role of chemical history in the sensitivity to patterned ELF-MF.

Interactions between ELF-MF and addictive psychoactive substances also exist. In two related experiments, the ability of these fields to influence cocaine response was shown. In one case, a 60 Hz, 2 milliT MF was found to potentiate the lethal effects of cocaine while in another a 4 Hz, 2 milliT field was found to influence the seizures and premorbid behaviours induced by cocaine [61]. Further, 50 Hz MF have been shown to blunt the locomotor response to amphetamine with prolonged exposures [42]. Moderate but not low or high doses of ampethamine also appear to interact with this configuration [112]. In these experiments, catecholamine involvement was strongly suspected and exposure period was a critical factor.

The aforementioned treatment of Parkinson's disease with ELF-MF is another promising application for a disease that may be co-promoted by environmental exposures to neurotoxins. Sandyk has found that application of a complex patterned, 7.5 picoT MF field with a median frequency in the 2 Hz range suppressed the side effects of Parkinson medication levodopa. He hypothesized this occurred through melatonin- or opiate-mediated [136, 142] mechanisms. The treatments employed by Sandyk were also effective on their own [135] and may ultimately reduce the requirement for medication. The treatment of multiple sclerosis and its animal models (experimental allergic encephalomyelitis) may also be improved by brief but repeated, particularly nocturnal, exposures to extremely weak ELF-MF [63, 110].

Rational “tuning” of the applied ELF-MF to the intensity of the components of the local geomagnetic field, based upon models of physical resonance, may be essential for “opening the gateways” for maximum synergisms. Daily exposures to a theta burst-patterned, 200 to 500 nanoT, MF presented once every 4 s markedly enhanced rats’ capacities to discern a local static anomaly (185 microT) superimposed upon a mean background of 57 microT (SD=23 microT). The congruence was based upon a theory of “progressive polynomial resonance.” Vorobyov [171] found that rats exposed to a more traditional Ca^2+^-tuned static/time-varying field at 20.9 microT with a 48 Hz (the third harmonic of the cyclotron 16-Hz frequency) variation displayed increases in electroencephalographic power within the central frequencies of about 6 Hz, 10 Hz, 14 Hz, and 16 Hz. Injection of morphine (10 mg/kg) combined with MF produced marked changes in the profile compared to those evoked by morphine alone. The MF eliminated the effect of intracebroventricular morphine at 2 Hz, decreased the power in the 6.6 Hz to 7.5 Hz band and increased the power within the 12.3 Hz to 20.3 Hz band. Further, the MF interacted powerfully with intraperitoneal morphine injection.

### b. Extremely High Frequency Field (VHF) Interactions with Drugs 

Since the advent of communications technology utilizing VHF (MHz-GHz range) there have been many investigations into biological consequences of VHF exposure. Microwave cooking appliances, radar, and mobile communications devices are among the most prevalent of technologies associated with MW frequencies (300 MHz-300 GHz). A wide spectrum of biological and behavioral effects [20, 21, 43] have resulted from MW exposure; these effects are thought to be short-term and of rapid onset. While many positive effects have been reported, their replication has encountered difficulty. For example, the early results of Lai, Horita and Guy [75] showed an affect of MW on radial maze behaviour, but later work by Cobb and colleagues [16] and Cassel and colleagues [11] utilizing relatively slight differences in method did not.

In several cases, VHF-MF have been shown to operate in synergy with drugs. In one of the earliest investigations into drug-MW interactions, Thomas, Burch and Yeandle [163] showed a potentiation of the effect of anxiolytic chlordiazepoxide (at clinical dosages) on fixed interval behaviour for food reinforcement by a 2.8 GHz, 1 mW/cm^2^pulsed MW. On its own, the MW was ineffective. This relationship between MW and drug efficacy could not be extended to other anxiolytics such as diazepam or chlorpromazine [164]. While it is unlikely that chlorpromazine interacts with MW to affect fixed interval behaviour, it does influence the hyperthermia induced by stronger (60 mW/cm^2^) MW [44]. As these MW patterns are scarcely encountered, they do not pose an occupational hazard but are of interest in understanding the mechanisms by which MW influences the biological system.

Drug-MW synergies have also been shown to work in reverse, with MW exposure enhancing the effect of normally inefficient drugs. Quock, Kouchich, Ishii and Lange [120] showed that co-administration of the antidopaminergic agent domperidone and MW at 2.45 GHz, 20 mW/cm^2^ for 10 min could counteract the effects of DA agonist apomorphine on stereotypic climbing behaviour in mice. Neither the drug nor the MW in isolation had an effect on climbing behaviour. In earlier experiments, the ability of applied MW to facilitate methylatropine antagonism of the chlolimimetic drug effects by muscarinic acetylcholine agonists pilocarpine and oxytremorine [118] was noted even though methylatropine alone had little effect.

Just as the parameters of the MW strongly influence the outcome of drug-MW experiments, so too can the parameters of the drug regime. In later work with chlordiazepoxide, Quock, Klauenberg, Hurt and Merritt [119] found that selective interactions existed between the applied drug and MW treatment. In their experiment, only acute exposure to very high frequency, very strong MW (4.7 GHz, 36 W/kg, applied for 300 sec) counteracted the effects of chlordiazepoxide on rearing and steps ascended in the mouse staircase test.

MW have potent interactions with other clinically relevant drugs. In the first of a series of experiments, Lai and colleagues showed the ability of acute exposure to 2.45 GHz pulsed waves of between 3-6 mW/cm^2^ to influence the stereotypy and thermal responses induced in rats by psychoactive compounds [73]. MW pulses of this configuration were shown to enhance apomorphine-mediated hyperthermia and stereotypy and attenuate amphetamine-induced hyperthermia. Other effects were found when chlorpromazine and 5.6 GHz EM were applied [45]. Exacerbation of morphine-induced catalepsy and lethality was also seen with MW treatment at certain dosages of the drug. Modulated VHF-MF are known to potentiate the hypnogenic effect of hexenal and may lower the required dose of haloperidol to induce catalepsy. Lasting effects on haloperidol were found 24h after exposure in the open field test [149].

Lai and others have also shown that their MW pattern can influence the rate of but not the final level of the hypothermic response to injected ethanol [74]. Consumption of an ethanol-fluid solution after exposure was also found to be effected by the combined treatment. Other experiments have shown an ability of ethanol to attenuate MW-induced changes in blood brain barrier permeability [99] which were found to be short-term responses to acute exposure only [39]. The results obtained by Cosquer and others [19] show no effect of MW on scopolamine methylbromide administration and suggest the likelihood of a universal increase in blood brain barrier permeability to all substances is low.

Ultra-wide band radiation emitted by technology (UWB) is also of concern, but the recency and scarcity of it has left little room for investigation. While only mild behavioral deficits arise from UWB exposure during development [17] few interactions with medication have been examined. It is known that UWB has no effect on pentylenetetrazol induced convulsions and does not appear to possess anticonvulsant properties [95]. Acute UWB can reduce the hyperactivity induced by nitric oxide synthase inhibitors in mice [146] but seems to have little effect on nociception [145, 146]. No interactions with morphine were found [145] which is surprising given the results found with MW and other VHF-MF and suggests UWB may exert its influence through different mechanisms.

### c. Static Magnetic Field (SMF) Interactions with Drugs 

The biological effects of SMF have always been of interest but became a serious concern when use of Magnetic Resonance Imaging (MRI) technology in diagnosis became common. The intensity to which the individual is exposed in an MRI is many thousands of times stronger (1 T to 5 T) then that for incidental ELF exposures. Most SMF studies use intensities within this range. The physical properties of these SMF are likewise considerably different from both ELF- and VHF-MF. While MRI technology appears safe and the influence of even intense SMF is weak, there are several studies which suggest strong, potentially negative effects and there is a need for replication of these positive findings [12, 24, 144]. Complex interactions may also occur between SMF and time-varying fields in the environment [176, 177].

Compared to the body of literature supporting ELF- and VHF-MF drug interactions, investigations examining the effects of SMF on drugs are scarce. Research into the potential of strong SMF to increase effectiveness of anti-cancer drugs, particularly in cell lines, is a notable exception [13, 107, 108, 165]. A review of the role of SMF in anti-cancer therapy is presented by Gray, Frith and Parker [38]. The effect of SMF on medication of epilepsy [93] and modulation of vasculature [36, 102-106] comprise the remaining portion of SMF-drug studies.

The interactions between SMF and other drugs, such as psychotropics, have received little attention in recent literature, but have been reported. Kholodov and others [60] reported very specific synergisms between pharmacological agents and a 50 milliT horizontal SMF that by itself produced a three fold increase in the percentage of spindles and slow waves within the hypothalamus, sensorimotor cortices, thalamus, caudate, midbrain reticular system and hippocampus of rabbits. Intravenous adrenaline (.03 mg/kg) produced a marked increase in this response while the sedative nembutal (13 mg/kg) and the antipsychotic aminasine (12 mg/kg) sharply decreased the response. The direct effect of the SMF on the brain was supported by the absence of this electrophysiological response when the posterior ventrolateral thalamus or posterior hypothalamus was lesioned prior to exposure. Lesion of the cerebral cortices had no effect.

Effects of SMF on pharmacological modulation of vasculature are well described and have much potential. Recently, Gmitrov and Ohkubo [36] showed an ability of the Ca^2+^ channel blocker verapamil to reverse 0.35 T SMF-induced increases in baroflex receptor sensitivity. Their study also showed that verapamil could reverse the decreases in baroflex receptor sensitivity caused by geomagnetic disturbances. Their variations are within the mHz range with peak-to- peak variations primarily between 20 nT (a frequent bioeffective threshold) and several 100 nT over durations of 1 ksec to 100 ksec. Other agents acting on calcium channels have similar synergies with drugs. The vascular effects of 2 mg/kg L-type Ca^2+^ channel blocker nicardipine were also potentiated by an mT range SMF [105, 106] even though the SMF was not very effective alone [105]. SMF have also been shown to reverse norepinephrine-mediated increases in blood pressure [102-104]. The ability of clinically silent fields to reverse the effects of L-NAME on vasculature is also known [103].

Interactions with epilepsy medication also exist but are only beginning to be uncovered. The potential anticonvulsant properties of SMF may also interact with phenytoin [93] to reduce audiogenic seizures in DBA/2 mice. The effects of both SMF and ELF-MF in seizure models suggest tuned MF may non-invasively enhance medication of the disorder.

## IV. MECHANISMS AND PROCESSES 

The general pattern of results that emerges from this review is that MF affect opiate, cholinergic, serotonergic and dopaminergic systems as well as the gaseous neurotransmitter nitric oxide. However, there are two limitations of this summary. First, the systems studied are more likely to reflect convenience rather than concept as well as the contemporary consensus among researchers. Second, the generalization that "magnetic fields affect cholinergic pathways" is likely to be as over-inclusive as the statement "drugs affect cholinergic pathways". In the latter case, without specifying the specific structure of the drug, its dosage, its route of administration, and perhaps even its vehicle, generalized statements are meaningless. Analogously, multiple parameters of an MF determine its effect, mechanism, and interaction, if any, with pharmacological systems.

Electromagnetism is the only natural force that can affect matter at the various levels of discourse that have been arbitrarily defined by scientific inquiry. Consequently the application of appropriately patterned MF can produce changes in behaviour, organ weights, tissue activity, cellular function, molecular reactions, and even the characteristics of the electron and proton chains involved with these reactions [1]. This multi-leveled impact will ultimately allow an independent reference point by which the levels of scientific discourse are interrelated and could either replace or certainly supplement the contemporary model of linear reductionism. By observing the changes evoked by the same applied MF within different levels of discourse perhaps a rational equivalence of response can be discerned that would allow the "translation" of an effect within molecular signaling pathways to, for example, behavioral alterations.

This multiplicity of potential responses might explain, more than discipline-specific models of the phenomenon, a rationale for why many hypotheses for MF effects may be equally correct but only valid within their own particular level of space. Given the complexity of living systems and the parallel processing that is evident, particularly within the multitude of neurotransmitter systems, there may be a myriad of physical changes within the levels of discourse that respond to MF. The pursuit of a singular mechanism may be as elusive as attempting to explain a phenomenon that is defined only by a multiple regression equation containing a dozen independent variables with diminishing partial regression coefficients, with a single bivariate plot.

The effect sizes of MF treatments, that is the amount of variance in the dependent variable explained or accommodated by the treatment, are within the range of 10% to 20% for most neurochemical studies. This low magnitude suggests the powerful role of other mediating variables or the effect of MF upon only a subset of molecules within "the same" pathways. Considering the consequences of realizing the existence of isoenzymes, isomers, and electron subpopulations upon neuropharmacology, the assumption that an applied magnetic field affects all molecules of the same species "identically" may not be valid. On the other hand the effect sizes for most of the analgesia studies involving the whole organism and the interactions shown in MF-drug studies are often within the range of 40% to 50%. This strength suggests a closer causal proximity to the temporal-spatial processes that produced the effect. In all of these studies, however, the effect sizes seldom approach 90%, the size typical of ligand-receptor binding effects or other physicochemical reactions in which there is a linear causal connection between the stimulus and the response. The role of the individual may also be important; the possibility that individual hypersensitivity to MF may exist, in a manner similar to allergies for certain proteins, as an explanation for this variability [83] is under investigation.

Mechanisms by which MF effect living systems, and hence through which pharmacological agents could interact, have focused upon variants of Faraday's Law, in that "a changing magnetic field is associated with a changing electric field." Five of the most likely electric, magnetic or synergistic mechanisms to examine the nature of magnetic field transduction in biological systems have been proposed [29] and each has its own support within the literature. Other notable models exist [32] which explain the replicable effects of MF on cellular physiology [4, 125, 128, 150, 178], particularly the dynamic interactions with blood flow and microvasculature [92].

Usually mechanisms are based upon the temporal symmetry (either sinusoidal waves or square waves, for example) of the applied MF. The classic arguments against weak (< 1 microT) MF affecting cells are usually derived from the argument that the energies involved would not be sufficient to compensate for random thermal agitation (the Boltzman "boundary"). Similarly, theoretical arguments have been published against the effects of VHF-MF on the organism at low incident power densities (<  15 mW/cm^2^), yet many studies demonstrate positive effects at this range. The continued demonstration of subtle yet significant behavioral and neurochemical changes in spite of these theoretical impossibilities suggests that current models may be inadequate.

Sensitivity to small variations in geomagnetic activity and shielding from the geomagnetic field [22] are known to effect behaviour in animals, possibly because they evolved within this environment. The wave amplitudes produced by geomagnetic phenomena, such as micropulsations, and atmospheric phenomena, such as whistlers and spherics can be shown to induce inside cells currents of the same magnitude (1-30 pA) as the currents generated by the cells themselves [27]. Even conservative estimates [ 33] show that a 1 microV induced membrane potential can be detected after 10 msec by an ensemble of less than 10^8^ ion channels. Liboff and others [77, 79] showed the threshold intensity to affect DNA synthesis by a wide range of sinusoidal MF (15 Hz to 4 kHz). The effect, which was maximum during the middle of the S-phase of the cell cycle, was independent of the time derivative and suggested a mechanism other than Faraday's Law.

The concept of MF effects being enhanced by the temporal congruence between the applied MF and intrinsic variations of the target molecule, cell, cell aggregate, or even the organism assumes strong magnetic fields are not required. According to Persinger [110], Jacobson [40, 41] and Sandyk [137] even pT to nT magnetic fields are effective with appropriate resonance as a function of the mass of the target molecule. There is strong mathematical evidence that MF absorption can occur in stochastic cellular systems through enhanced detection in ion channels and by calcium oscillators [134].

That MF ultimately affect ions would accommodate for the diffuse nature of their reported effects across levels of scientific discourse. For example, if Ca^2+^ ions are primarily affected, as first hypothesized by Ludwig [82], and later verified by Adey and others [1, 7], then this modification percolated through larger and larger organismic space could explain the positive effects of magnetic fields upon reducing depression [5] due to alterations in Ca^2+^ signaling [173]. Voltage gated Ca^2+^ L-type channels have been shown to mediate the stimulation of neurite growth in cultured chromaffin cells by 60 Hz (0.6 milliT) fields because the blocker nifedipine eliminated this response [98]. These channels may play in a role MF-induced analgesia [57] and vascular changes [105, 106] and may facilitate absorption of EM and its information by stochastic processes [134].

As mentioned previously, reconciling MF-induced variations in NO activity with the sequelae of MF exposure is difficult. The diffuse pattern of activation might suggest a cascade of intracellular events with the intermediate participation of ions such as Ca^2+^ and their associated cellular machinery. However, there will be a need to accumulate additional experiments controlling for these variables. The characterization of the role of these in the generation of MF responses requires a comprehensive body of literature that allows for comparison of the role of these variables in MF responses at different chronological intervals.

## V. IMPLICATIONS 

The observation that weak physiologically patterned MF can interact with the effects of pharmacological agents to alter or to significantly increase the potency of pharmacotherapy has powerful implications for this century. As the usage of both pharmacological agents and electromagnetic fields for communication systems and appliances increases within Western civilization, synergistic interactions must occur. In several studies the interaction between intensities of pulsed or physiologically-patterned MF potentially present within the environment and frequently employed pharmacological agents such as clonidine [85] and chlorodiazepoxide [163] produced effects that were several magnitudes greater than those from the application of simply the drug itself.

If MF-drug synergisms are as specific as the simple sequestering found between ligands and high affinity receptor subtypes, then weak (1 microT, at 42.5 Hz for the proton) and very, very weak (1 nT, at 28 Hz for the electron), appropriately patterned MF could produce unanticipated exacerbations of neuropharmacological responses. The presence of these interactions, and the inability to resolve them mechanistically, reflects a need for an integrated, interdisciplinary approach in future bioelectromagnetics research. Pharmacology has yet to apply the models of quantum mechanics and fine-structure electromagnetism to drug effects. For example, NO, which is involved in a multitude of MF responses, is also the only free molecule whose magnetic susceptibility does not conform to the "spin-only" formula. NO is a classic example of a molecule that deviates from Curie's Law because the multiplets of the atomic spectra interval are comparable with the kT boundary [170]. Elicitation of quantum phenomena by hereto unexpected synergisms between "routine" pharmacological agents and specifically designed magnetic patterns could result in macroscopic changes with emergent properties with unanticipated potency.

The employment of patterned magnetic fields to discern the presence of pharmacological compounds has numerous applications. One historical example in neuropharmacology was the display of conspicuous vascular and visceral responses to disulfiram if the person's blood level contained ethanol but no response if there was no ethanol. A similar synergism may exist with magnetic fields. Many years ago, Kholodov [60] showed that static magnetic fields could not be detected by the average person. However during states of hypnosis, or after consumption of mescaline, modifications of the visual images seen by volunteers when MF were applied could be clearly discerned. Persinger [109] reported that during casual intoxication by Lysergic Acid Diethylamide (LSD-25) participants reliably reported specific colors emerging around rotating MF within a completely darkened room. These "colors" were not reported when the field was present but the subjects had not consumed LSD. Such reactions, while not intensively studied, point to possible influences of MF on psychotropic drug states yet to be characterized [133].

The understanding of neuropharmacological synergisms with MF within the brain also requires a greater empirical understanding of the specific, endogenous temporal patterns of electrophysiological activity. Specific changes in the temporal pattern of effective stimuli that produce long term potential (LTP), a phenomenon once considered to be relatively homogenous, vary across different layers of the entorhinal cortices in the rat [159]. This suggests that appropriately patterned MF may maximally interact with pharmacological reactions only in those brain volumes that contain resonant patterns. As well as different molecules being effected by MF, different regions may be effected as well.

There are two discrete brain regions within which pharmacological agents and physiologically-patterned MF might interact. The first is the pineal organ which contains the synthesizing enzymes for melatonin as well as copious concentrations of Ca^2+^ both of which can be affected by MF whose frequencies coincide with the ion's resonance [76]. The precise afferent neuroanatomy of this gland in the human is still not clear, as indicated by the recent report [156] of a new paired tract that can be traced to the pretectal area. The role of the pineal gland in MF responses may be strongly affected by the species studied [126, 127, 166].

The second area involves the hippocampus and limbic system. This region of the brain, in conjunction with the amygdala, shows a lower threshold for paroxysmal activity than any other structure. The neurons located within this area are prone to phase coupling [172] and amplification with large volumes of the entire structure. Additionally, the hippocampal formation contains magnetite and related magnetic material sufficient to allow epileptiform activity to be evoked in epileptic patients by applied magnetic fields of only 1 milliT [26] Altered brain metabolism of iron has been considered a significant contributor to neurodegenerative diseases because the release of iron ions from injured brain cells stimulates free radical reactions [35]. Pharmacological compounds that modulate the functional susceptibility of these substances, which can exist in concentrations greater than pg/g of tissue, might contribute to significant synergisms.
